# Long-Term Monitoring of Cardiac Involvement under Migalastat Treatment Using Magnetic Resonance Tomography in Fabry Disease

**DOI:** 10.3390/life13051213

**Published:** 2023-05-19

**Authors:** Constantin Gatterer, Dietrich Beitzke, Senta Graf, Max Lenz, Gere Sunder-Plassmann, Christopher Mann, Markus Ponleitner, Robert Manka, Daniel Fritschi, Pierre-Alexandre Krayenbuehl, Philipp Kamm, Olivier Dormond, Frédéric Barbey, Pierre Monney, Albina Nowak

**Affiliations:** 1 Department of Medicine II, Division of Cardiology, Medical University of Vienna, 1090 Vienna, Austria; constantin.gatterer@meduniwien.ac.at (C.G.);; 2 Department of Biomedical Imaging and Image-Guided Therapy, Medical University of Vienna, 1090 Vienna, Austria; 3 Department of Medicine III, Division of Nephrology and Dialysis, Medical University of Vienna, 1090 Vienna, Austria; 4 Department of Neurology, Medical University of Vienna, 1090 Vienna, Austria; 5 Institute of Diagnostic and Interventional Radiology and Department of Cardiology, University Hospital Zurich, University of Zurich, 8091 Zurich, Switzerland; 6 University Heart Center, University Hospital Zurich, 8091 Zurich, Switzerland; 7 Department of Endocrinology and Clinical Nutrition, University Hospital Zurich and University of Zurich, 8091 Zurich, Switzerland; 8 Radiology Department, Spital Langenthal, 4900 Langenthal, Switzerland; 9 Department of Immunology, Centre Hospitalier Universitaire Vaudois (CHUV) and University of Lausanne, 1011 Lausanne, Switzerland; 10 Department of Cardiology, Centre Hospitalier Universitaire Vaudois (CHUV) and University of Lausanne, 1011 Lausanne, Switzerland; pierre.monney@chuv.ch; 11 Division of Internal Medicine, Psychiatric University Hospital Zurich, 8008 Zurich, Switzerland

**Keywords:** Fabry disease, cardiac imaging, cardiac magnetic resonance, chaperone, migalastat, cardiomyopathy, lysosomal storage, left ventricular hypertrophy, *GLA*, genetic disease

## Abstract

**Background:** Fabry cardiomyopathy is characterized by left ventricular hypertrophy, myocardial fibrosis, arrhythmia, and premature death. Treatment with migalastat, an oral pharmacological chaperone, was associated with a stabilization of cardiac biomarkers and a reduction in left ventricular mass index, as measured by echocardiography. A recent study, using cardiac magnetic resonance (CMR) as the gold standard, found a stable course of myocardial involvement after 18 months of treatment with migalastat. Our study aimed to provide long-term CMR data for the treatment with migalastat. **Methods:** A total of 11 females and four males with pathogenic amenable *GLA* mutations were treated with migalastat and underwent 1.5T CMR imaging for routine treatment effect monitoring. The main outcome was a long-term myocardial structural change, reflected by CMR. **Results:** After migalastat treatment initiation, left ventricular mass index, end diastolic volume, interventricular septal thickness, posterior wall thickness, estimated glomerular filtration rate, and plasma lyso-Gb3 remained stable during the median follow-up time of 34 months (min.: 25; max.: 47). The T1 relaxation times, reflecting glycosphingolipid accumulation and subsequent processes up to fibrosis, fluctuated over the time without a clear trend. No new onset of late gadolinium enhancement (LGE) areas, reflecting local fibrosis or scar formation of the myocardium, could be detected. However, patients with initially present LGE showed an increase in LGE as a percentage of left ventricular mass. The median α-galactosidase A enzymatic activity increased from 37.3% (IQR 5.88–89.3) to 105% (IQR 37.2–177) of the lower limit of the respective reference level (*p* = 0.005). **Conclusion:** Our study confirms an overall stable course of LVMi in patients with FD, treated with migalastat. However, individual patients may experience disease progression, especially those who present with fibrosis of the myocardium already at the time of therapy initiation. Thus, a regular treatment re-evaluation including CMR is needed to provide the optimal management for each patient.

## 1. Introduction

Fabry disease (FD) is a rare X-linked lysosomal storage disease resulting from pathogenic *GLA*-gene mutations leading to the markedly reduced or absent activity of the enzyme α-galactosidase A (α-Gal A) and subsequent glycosphingolipid accumulation in lysosomes. There are two major phenotypes, classic and late-onset [1]. In males, the classic phenotype is more severe due to low (<3%) residual α-Gal A activity, with early symptoms including acroparesthesia, angiokeratoma, corneal opacities, and hypohidrosis [2]. The continuous sphingolipid deposition gradually leads to cardiomyopathy, chronic nephropathy, and premature strokes [2]. In heterozygous females, α-Gal A activity can range from low to normal due to random X-chromosomal inactivation [3]. Fabry cardiomyopathy, characterized by increasing left ventricular hypertrophy, and the development of myocardial fibrosis are frequently accompanied by progressive chronic kidney disease and premature strokes. The disease leads to a reduction in life expectancy of about 20 years in males and 15 years in females, if untreated [4,5,6].

Currently, two types of specific therapies are available for the treatment of FD: intravenous recombinant enzyme replacement therapy (ERT), every other week (agalsidase alfa or agalsidase beta, both approved by the European Medicines Agency in 2001), and, more recently approved (2016 in the European Union and 2017 in Switzerland), the oral pharmacological chaperone migalastat every other day [7,8,9]. Studies on the clinical effects of migalastat, including the randomized phase III trials ATTRACT and FACETS, as well as “real-world data”, showed promising results in terms of stabilization or improvement of left ventricular hypertrophy [10,11,12,13,14]. However, 2D transthoracic echocardiography (TTE) was used for the quantification of left ventricular mass (LVMi) in these studies. Previous studies had demonstrated that TTE might not be the preferred method for quantifying LVMi because of its pronounced inter- and intra-observer variability, and therefore, greater patient numbers would be needed to detect a significant change [15,16,17]. Recently, Camporeale et al. also provided important CMR data, confirming a stable course of LVMi under treatment with migalastat in previously treatment-naïve patients [18]. Our study aimed to examine the evolution of Fabry cardiomyopathy under migalastat treatment in a “real-world” cohort of patients who were pre-treated with ERT and treatment-naïve patients, using cardiac magnetic resonance (CMR) as the current gold standard method.

## 2. Materials and Methods

This retrospective multinational study complies with the declaration of Helsinki and was approved by the respective local institutional review boards. Written informed consent was obtained from all patients. All consecutive adult patients with pathogenic amenable *GLA* mutations on migalastat treatment who underwent ≥1 annual CMR as part of therapy monitoring at the specified Fabry centers in Zurich (n = 6) and Lausanne (n = 2), Switzerland and Vienna (n = 7), Austria, were included. Patients started treatment with migalastat between April 2017 and December 2020, and the last follow-up was censored in February 2023. CMR examinations of at least 6 months before the treatment with migalastat (“pre migalastat”) and <1 month before the initiation of migalastat (“treatment start”), and all follow-up CMR examinations, were analyzed. Examinations < 1 month prior to treatment start or the first under treatment with migalastat were defined as “baseline”. All CMR examinations were conducted according to the actual recommendations of the society of cardiovascular magnetic resonance imaging on 1.5T scanners for every series of follow-ups of a respective patient, except for one conducted on a 3T scanner [19]. LVMi, interventricular septal thickness (IVS), posterior wall thickness (PWT), left ventricular ejection fraction (EF), end-diastolic volume (EDV), ejection fraction (EF), late gadolinium enhancement (LGE) and septal T1 relaxation times before the application of the contrast medium were measured using specific CMR post-processing software (Medis Suite MR, Medis Medical Imaging, Leiden, Netherlands or Syngovia, Siemens Healthcare, Erlangen, Germany) according to current guidelines [20]. Left ventricular hypertrophy was defined as an LVMi, with papillary muscles excluded from mass, of >75g g/m^2^ for males and >59g g/m^2^ for females, according to current recommendations [21]. The extent of myocardial fibrosis detected by late gadolinium enhancement (LGE) was measured as a percentage of the left ventricular mass using the full width at half-maximum method in a dedicated software (Medis Suite MR, Medis Medical Imaging, Leiden, Netherlands or GT volume, Gyrotools, Zurich, Switzerland). Although the same software was used for all examinations of a respective patient, in order to avoid inter-software variability, the particular software and MRI vender were chosen depending on the respective center, thus leading to an impairment in the accuracy and inter-patient comparability.

In the present analysis, the clinical data and laboratory results were extracted from medical records.

α-Gal A activity was expressed as a percentage of the lower reference limit, based on the specifications of the local laboratories. α-Gal A activities were initially determined at the time of diagnosis and measured in peripheral leucocytes as part of therapy monitoring. The α-Gal A sampling was random and not related to migalastat administration. In parallel, lyso-Gb3 levels in dried blood spots (DBS) were determined. Pathogenic mutations were assigned as amenable or nonamenable to migalastat treatment based on the amenability table (http://www.galafoldamenabilitytable.com/hcp, accessed on 28 December 2021) provided by Amicus Therapeutics. Estimated glomerular filtration rate (eGFR) was calculated using the Chronic Kidney Disease Epidemiology Collaboration equation (CKD-EPI) [22].

Statistical analyses were performed using SPSS V26 (IBM, Armonk, NY, USA). Descriptive statistics were used for demographics and clinical parameters. Categorical variables were expressed as proportions, and continuous variables as medians, interquartile ranges (IQR) or mean ± standard deviation. Wilcoxon tests were used to compare the non-normally distributed cardiac and laboratory variables between the baseline and individual latest follow-up examinations, and the t-test was used for normally distributed variables. All statistical tests were two-sided. A *p*-value below 0.05 was considered significant.

## 3. Results

Overall, 16 patients with an amenable *GLA*-mutation for treatment with migalastat were initially included in the study. One patient was switched back to ERT before the first CMR follow-up due to an increase in proteinuria, and was excluded from the analysis. T1-mapping sequences from one patient were excluded since the baseline examination was performed on a 3T scanner. In total, 15 patients (11 females) with a median follow-up time of 34 months (min: 25, max.: 47) were analyzed. The median age at therapy initiation was 54 years (min.: 29, max.: 75). Seven patients were previously treated with ERT, and eight patients were treatment-naïve. In total, 10 patients (6 females and all males) presented with left ventricular hypertrophy (LVH) at the time of therapy initiation. Seven patients (46.7%) had LGE detected at baseline. Baseline patients’ characteristics are shown in Table 1.

**Figure 1:**
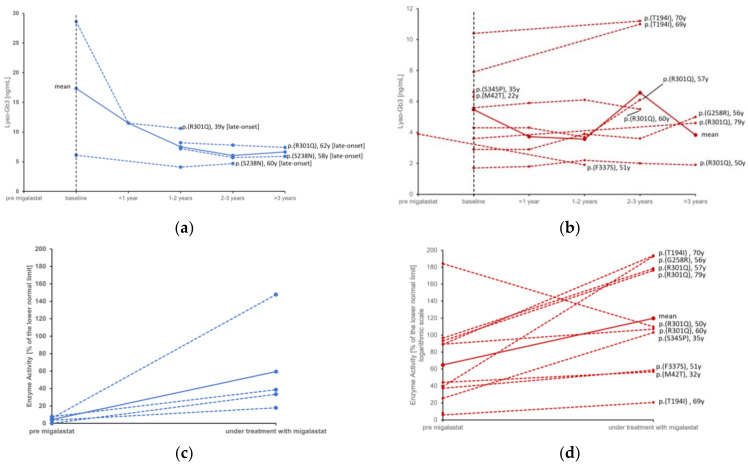
Individual courses of lyso-Gb3 in males (**a**) compared to females (**b**) and enzyme activity before and during treatment with migalastat in males (**c**) compared to females (**d**). Red = female, blue = male. Continuous lines represent the mean value. [late-onset] = late onset phenotype of Fabry disease; [classic] = classic phenotype of Fabry disease. Baseline = “treatment start”.

During migalastat treatment, the median α-Gal A activity significantly increased from 37.3% (IQR: 5.88–89.3) to 105% (IQR: 37–177 *p* = 0.005), although individual patients showed little or no improvement in enzyme activity (Figure 1). Conversely, Lyso-Gb3 showed a non-significant trend towards reduction, mainly in male patients.

**Figure 2:**
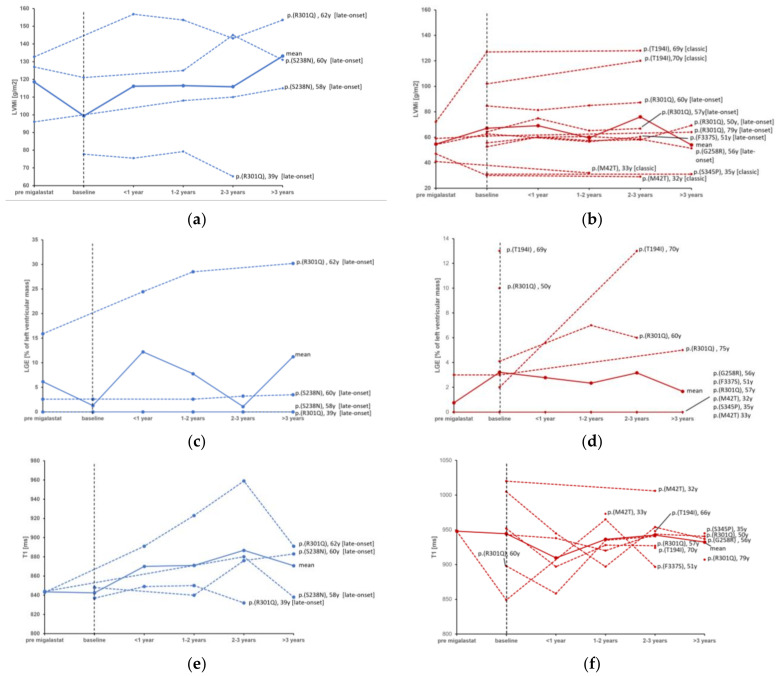
Individual courses of CMR-measured LVMi in males (**a**) compared to female patients (**b**), LGE in males (**c**) compared to females (**d**) and T1 relaxation times in males (**e**) compared to females (**f**) before and during treatment with migalastat. Red = female, blue = male. Continuous lines represent the mean value. [late-onset] = late onset phenotype of Fabry disease; [classic] = classic phenotype of Fabry disease. LVMi, left ventricular mass indexed; LGE, late gadolinium enhancement. Baseline = “treatment start”.

During the observational period, between the individual baseline or earliest follow-up CMR under migalastat treatment and the individual latest follow-up, there were no significant changes in LVMi, IVS, PWT or LVEDV in the total cohort or in the subgroups of patients with LVH at baseline or male/female patients. Median values of CMR parameters at baseline and the individual latest follow-up are displayed in Table 2. The individual analysis of LVMi (Figure 2) shows an overall stable course of Fabry cardiomyopathy in most, predominantly female, patients. However, some patients showed a trend towards the progression of LVMi despite therapy with migalastat, such as two male (one presenting with LGE, one without) patients with the late onset mutation c.713G>A p.(S238N) aged 58 and 60 years, respectively, and a 70-year-old female with the classic mutation c.581C>T p.(T194I), who also presented with progressive LGE, despite being the only patient receiving co-medication with acetylsalicylic acid (ASA). In contrast, a stabilization of Fabry cardiomyopathy was observed in a 69-year-old female with the same classic mutation c.581C>T p.(T194I). Another 46-year-old female patient carrying the c.902G>A p.(R301Q) late-onset mutation with LGE already at baseline showed a mild increase in LVMi. No correlation could be found between the change in α-Gal A activity and the course of LVMi.

Patients with no LGE at baseline did not develop new-onset LGE, whereas five of the total seven patients with pre-existing LGE, in whom LGE was measured during follow-up, displayed a significant increase in myocardial fibrosis from 3% (2.3–14.3%) of the LV mass to 6% (4.24–21.6%) under therapy with migalastat (Figure 2). The median T1 relaxation time varied over time, though no clear trend could be observed.

Median and individual left ventricular EF were normal at the beginning of the treatment, and did not change significantly during the observational period.

The individual eGFR showed variations between the different examinations, but remained globally stable. An increase in the urinary protein to creatinine ratio was observed in a 31- and a 28-year-old female with the classic mutations c.125T>C p.(M42T) and c.1033T>C p.(S345P), respectively. Individual eGFR courses can be found in the Supplemental Figure S1.

## 4. Discussion

During long-term detailed patient monitoring, migalastat led to increased enzyme activity levels and a stable course of CMR and laboratory markers in our cohort of patients with different stages of Fabry disease.

A reduction in echocardiographically measured LVMi, quantifying LVH—one of the main characteristics of Fabry cardiomyopathy and a risk factor for cardiac events—was reported in the phase III trials, with a persistent effect in patients with LVH in the open-label extension study [9,13,14,23]. Further studies, including a recent collaboration of German Fabry disease centers, substantiated these initial findings by adding promising “real-world” data, and concluding that therapy with migalastat was generally safe and associated with a stabilization or improvement in LVH [10,12,24,25,26]. Very recently, the stable course of LVMi under migalastat was also confirmed by a study using CMR [18]. Indeed, we also found an overall stable course of CMR-measured LVMi and EF in patients with or without LVH at migalastat initiation, but we could not observe a reduction in LV mass under treatment. The detailed analysis of single patients’ disease courses revealed possible disease progression despite therapy with migalastat in male and female patients, thus highlighting the need for regular treatment monitoring, including CMR.

So far, the recently published MAIORA study by Camporeale et al. is the first to use CMR to evaluate the treatment effects of migalastat, as the previous studies relied on 2D echocardiographic-estimated LVMi to evaluate myocardial dimensions. Although actions were taken to reduce variability between the examinations, such as using a single-blinded investigator, 2D echocardiography may not be the preferred method for detecting differences in myocardial mass. In fact, it has been demonstrated that the number of patients needed to detect a difference of 10 g myocardial mass with echocardiography, with a beta error of 5%, lies between 152 and 626 [15,16,17]. As with most studies in the field of rare diseases, none of the previous studies regarding therapy with migalastat reached these patient numbers. The use of CMR allows a reduction in sample size, hence representing the method of choice for the quantification of LVH, especially in small cohorts [15,16,17].

**Figure 3:**
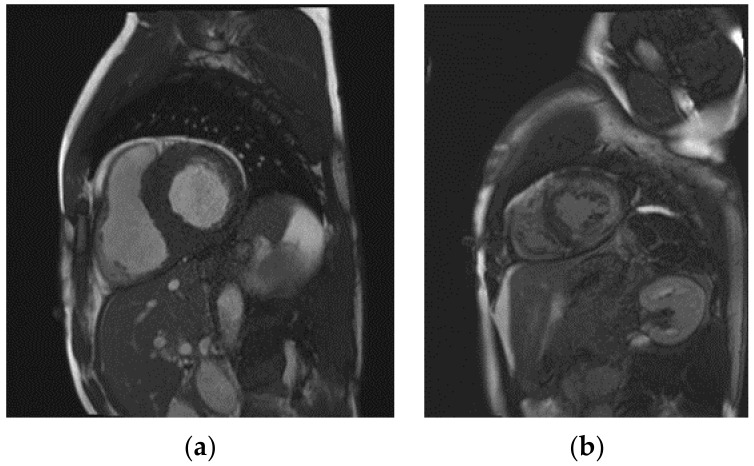
Exemplary cardiac magnetic resonance images of a Fabry disease patient with pronounced left ventricular hypertrophy (**a**) and an extensive area of late gadolinium enhancement (**b**).

Another important argument for using CMR to evaluate disease progression in FD is the detection of local fibrosis or scarring by LGE. So far, little is known about the course of LGE and its association with changes in myocardial wall thickness or LVMi, especially in Fabry disease. A study by Raman et al. recently found an association of LGE progression with myocardial wall thinning in patients with hypertrophic cardiomyopathy [27]. Although the process of myocardial glycosphingolipid storage and subsequent inflammatory responses in FD differs from the various mechanisms causing LVH in hypertrophic cardiomyopathy, previous studies showed a substantial clinical effect of LGE on the LVMi, as patients may suffer from an underestimated sudden cardiac death risk, based on a lower estimated degree of LVH [27,28]. Indeed, one of the patients analyzed in our study showed a reduction in posterior wall thickness, accompanied by an increase in LGE, and eventually received an implantable cardioverter–defibrillator. However, treatment with migalastat may limit the initial formation of fibrosis, as no new-onset LGE could be observed in our cohort. On the other hand, our study confirmed another finding of the MAIORA study, with a significant increase in LGE in those with pre-existing fibrotic areas of the myocardium [18]. An explanation for this progression, despite specific therapy, may lie within the inflammatory and other cellular processes that are caused by glycosphingolipid deposition and lead to LGE. Fabry-specific treatment may not be able to immediately terminate these cascades of cell damage. Exemplary CMR images of a patient with extensive LGE can be found in Figure 3.

In the past decade, measuring T1 relaxation times of the myocardium became a cornerstone in the evaluation of cardiac disease involvement in Fabry disease, as it is proposed to reflect the different stages, including glycosphingolipid accumulation within the myocardium, as well as the subsequent inflammation and fibrosis seen in late stages, with initially low T1, followed by a pseudo-normalization [29,30]. The heterogeneous distribution of disease stages within our study cohort may be the reason for the variation in T1 without a clear trend after treatment initiation.

A significant increase in enzymatic α-Gal A activity could be detected after treatment initiation, although not in all individual patients. This might be the effect of “only slightly migalastat-amenable” *GLA* variants, as discussed by Lenders et al. [25]. The treatment effect in these variants is unclear, although this may be a reason for the progression of LVH and LGE in some patients. However, we did not find a correlation between the measures of α-Gal A activity and the course of LVMI or LGE.

Additionally, kidney function, assessed by eGFR using the CKD-EPI equation, remained stable during the observational period, supporting the findings of a post-hoc analysis of FACETS, ATTRACT, and the open-label extensions by Bichet et al., who found a long-term stabilization of kidney function in patients being treated with migalastat [11].

Regular monitoring including CMR is necessary in migalastat-treated patients, particularly for those with LGE, who may benefit from shorter monitoring intervals, because myocardial fibrosis could progress and lead to an impairment of left ventricular function and malignant arrhythmia. Accordingly, concomitant cardiac drug and device therapy should be optimized for each individual patient.

Recently, an enhancing effect on chaperon therapy was described for the widely used drug acetylsalicylic acid (ASA). The group of Monicelli et al. found that, in a cell model, the combination of migalastat and ASA led to a greater increase in α-Gal A activity and clearance of Gb3 and lyso-Gb3 than migalastat alone, while prolonging the enzyme-stabilizing effect [31]. The mechanism may be due to an interaction with the heat shock 70kDA protein 5 (HSPA5), also known as BiP, an endoplasmic reticulum chaperon, responsible for folding and quality control. In our study, only one patient was co-treated with ASA. Interestingly, this one female patient with the p.(T194I) variant who received ASA before treatment with migalastat experienced a significant increase in enzyme activity from 89 to 194% of the lower normal limit. However, she also suffered from cardiac disease progression after the observational period of 3 years. An increase in the LGE extent from 2 to 13% and LVMi from 102 to 120 g/m^2^ could be observed within 3 years, while lyso-Gb3 was only slightly elevated in comparison with the baseline value (10.4 vs. 11.2 ng/mL). Further concepts of a dual therapy include the use of proteostasis regulators, as well as combining ERT and chaperone therapy [32,33,34]. For ambroxol, another widely used drug that interferes with α-Gal A via a so-far elusive mechanism, contradictory studies can be found regarding its effect on enzyme activity [33,35]. However, none of the study participants were treated with ambroxole or any other possibly interfering drug such as rosiglitazone or bortezomib during or close to the treatment with migalastat, although unknown drug interactions cannot be excluded. Extensive clinical testing will be needed to provide reliable data on the co-therapy with migalastat-enhancing drugs.

The limitations of this study include the small sample size, with a high percentage of female patients and patients with late-onset variants. Additional to FD being a rare disease, only a proportion of patients are amenable to and qualified for treatment with migalastat, thus explaining the relatively small sample size. Given the fact that migalastat has been available in the European Union since 2016 and since 2017 in Switzerland, this study analyzes a long follow-up interval of a representable number of patients, who were consequently examined on the same software and 1.5T MRI scanners to minimize inter-device variability. Due to this study’s multicenter- and retrospective character, a different MRI scanner and software were used in the respective center, and not all patients underwent CMR examinations in the same interval; further, T1 estimation was unavailable in some cases, thus leading to missing values. Other causes for LGE, such as ischemic heart disease, were not systematically excluded if not clinically indicated. Nevertheless, previous studies found that coronary events may not be the main trigger for fibrosis in FD, and LGE areas were typically located in all patients [36,37]. Prospective long-term CMR studies with a larger cohort of FD patients are needed to provide further detailed evidence on the benefit of treatment with migalastat.

## 5. Conclusions

In summary, we found an overall stable course of CMR-measured left ventricular mass and function, as well as kidney function, in our cohort of patients with FD under treatment with migalastat. However, we could not detect a significant reduction in left ventricular mass. This is in contrast to previous echocardiography-based studies [9,10,12,14]. Individual patients of our study cohort, mainly those with LGE, but also patients with left ventricular hypertrophy only, suffered from disease progression despite specific therapy and therapy with a possibly α-Gal A-enhancing co-medication. Thus, regular treatment re-evaluations should be performed in all FD patients, and include CMR examinations, as echocardiography alone is not capable of detecting myocardial fibrosis or a slight deterioration of LVMi. The interval of visits and CMR examinations should be based on the disease manifestation.

## Figures and Tables

**Table 1:** Baseline characteristics.

	Overall (n = 15)	Male (n = 4)	Female (n = 11)
**Age at baseline (years)**	54 (37–59)	55 (46–58)	53 (32–66)
**Follow-up time (months)**	34 (26–43)	29.5 (26–40)	37 (26–43)
**Previous ERT**		3 (75)	4 (36)
**Left ventricular hypertrophy**	10 (66.7%)	4 (100%)	6 (54.5%)
**ACE-inhibitor/AT_1_-r. antagonists**	8 (53%)	1 (25%)	7 (64%)
**Acetylsalicylic acid**	1 (6.67%)	0 (0%)	1 (9.09%)
**Urinary protein to creatinine ratio**			
**<100 mg/g**	10 (66.7%)	1 (25%)	9 (81.82%)
**100–1000 mg/g**	4 (26.7%)	2 (50%)	2 (18.18%)
**>1000 mg/g**	1 (6.67%)	1 (25%)	0 (0%)
***GLA* mutations + (phenotype)**		c.902G>A p.(R301Q) (late-onset)	c.902G>A p.(R301Q (late-onset)
		c.902G>A p.(R301Q) (late-onset)	c.902G>A p.(R301Q) (late-onset)
		c.713G>A p.(S238N) (late-onset)	c.902G>A p.(R301Q) (late-onset)
		c.713G>A p.(S238N) (late-onset)	c.902G>A p.(R301Q) (late-onset)
			c.772G>A p.(G258R) (late-onset)
			c.1010T>C p.(F337S) (late-onset)
			c.125T>C p.(M42T) (classic)
			c.125T>C p.(M42T) (classic)
			c.1033T>C p.(S345P) (classic)
			c.581C>T p.(T194I) (classic)
			c.581C>T p.(T194I) (classic)

Values are given as median (interquartile range) or n (%). ERT, enzyme replacement therapy; ACE, angiotensin converting enzyme; AT1-r., Angiotensin II receptor, type AT1.

**Table 2:** Overview of functional and morphological CMR parameters during the observational period.

	Pre-Migalastat n = 8	Baseline n = 15	Follow-Up n = 15	*p*-Value for Change between Baseline and Follow-Up
**LVMi (g/m^2^)**	66 (51–111)	64.2 (52.5–108)	66.8 (51.4–120)	0.294
**IVS** **(mm)**	12 (7–15)	13 (10–14)	13 (11–15)	0.470
**PWT** **[mm]**	8.8 (7.5–12.5)	9 (8–11)	10 (8–13)	0.577
**LVEDV** **[ml]**	83 (77–107)	96 (66–108)	95 (76.8–121)	0.096
**LVEF** **[%]**	62 (59–71)	67 (60.6–76.9)	61 (58–72)	0.152
**LGE** **[%]**	n = 7 0 (0–3)	n = 15 0 (0–4.1)	n = 13 0 (0–5.5)	0.043
**T1** **[ms]**	n = 3 844 (843–948)	n = 14 916 (865–915)	n = 15 927 (891–945)	0.859
**eGFR** **[ml/min/1,73 m^2^]**	n = 9 99 (81–8-105)	n = 14 93.5 (78.7–102)	n = 15 90.48 (74–101)	0.074

Values are given as median (interquartile range). LVMi, left ventricular mass indexed; IVS, interventricular septum thickness; PWT, posterior wall thickness; LVEDV, left ventricular end diastolic volume; LVEF, left ventricular ejection fraction; LGE, late gadolinium enhancement; eGFR, estimated glomerular filtration rate.

## Data Availability

The datasets used and/or analyzed during the current study are available from the corresponding author on reasonable request.

## Supplementary Materials

The following supporting information can be downloaded at: https://www.mdpi.com/article/10.3390/life13051213/s1, Figure S1: Individual eGFR courses.
