# Supplementary material for: Long-Term Monitoring of Cardiac Involvement under Migalastat Treatment Using Magnetic Resonance Tomography in Fabry Disease
Source: Life (Basel). 2023 May 19;13(5):1213. doi: 10.3390/life13051213 (PMC10222787; doi:10.3390/life13051213)
Supplement: Supplementary file 1 [file life-13-01213-s001.zip › life-2308927-supplementary.pdf]

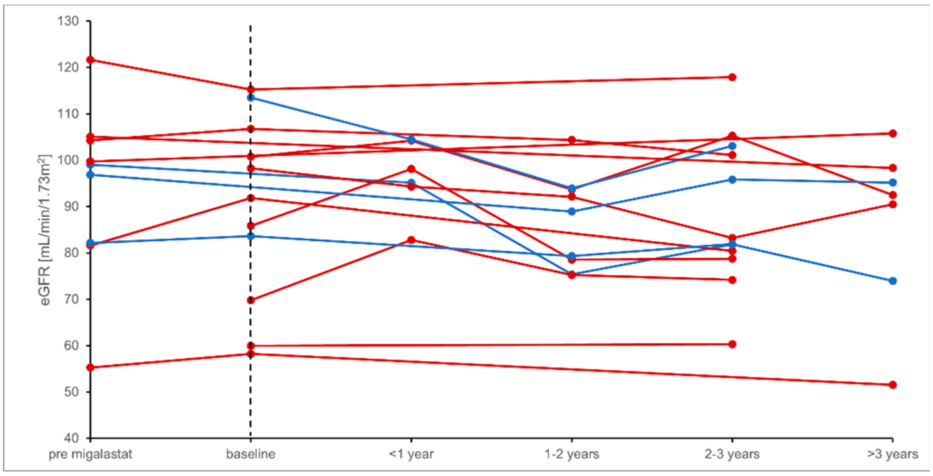


**Supplementary Figure S1.** Individual courses of eGFR males (blue) compared to female patients (red) eGFR, estimated glomerular filtration rate. Baseline = “treatment start”.
